# MGMT Expression Contributes to Temozolomide Resistance in H3K27M-Mutant Diffuse Midline Gliomas

**DOI:** 10.3389/fonc.2019.01568

**Published:** 2020-01-21

**Authors:** Hideaki Abe, Manabu Natsumeda, Masayasu Okada, Jun Watanabe, Yoshihiro Tsukamoto, Yu Kanemaru, Junichi Yoshimura, Makoto Oishi, Rintaro Hashizume, Akiyoshi Kakita, Yukihiko Fujii

**Affiliations:** ^1^Department of Neurosurgery, Brain Research Institute, Niigata University, Niigata, Japan; ^2^Department of Neurological Surgery, Northwestern University Feinberg School of Medicine, Chicago, IL, United States; ^3^Department of Biochemistry and Molecular Genetics, Northwestern University Feinberg School of Medicine, Chicago, IL, United States; ^4^Department of Pathology, Brain Research Institute, Niigata University, Niigata, Japan

**Keywords:** MGMT, diffuse midline gliomas, histone H3 mutation, epigenetics, DNA hypomethylation

## Abstract

Diffuse midline gliomas (DMGs) show resistance to many chemotherapeutic agents including temozolomide (TMZ). Histone gene mutations in DMGs trigger epigenetic changes including DNA hypomethylation, one of which is a frequent lack of O6-methyl-guanine-DNA methyltransferase (*MGMT*) promoter methylation, resulting in increased MGMT expression. We established the NGT16 cell line with *HIST1H3B* K27M and *ACVR1* G328E gene mutations from a DMG patient and used this cell line and other DMG cell lines with *H3F3A* gene mutation (SF7761, SF8628, JHH-DIPG1) to analyze *MGMT* promoter methylation, MGMT protein expression, and response to TMZ. Three out of 4 DMG cell lines (NGT16, SF8628, and JHH-DIPG1) had unmethylated *MGMT* promoter, increased MGMT expression, and showed resistance to TMZ treatment. SF7761 cells with *H3F3A* gene mutation showed *MGMT* promoter methylation, lacked MGMT expression, and sensitivity to TMZ treatment. NGT16 line showed response to ALK2 inhibitor K02288 treatment *in vitro*. We confirmed *in vitro* that MGMT expression contributes to TMZ resistance in DMG cell lines. There is an urgent need to develop new strategies to treat TMZ-resistant DMGs.

## Introduction

Diffuse midline gliomas (DMGs), comprised of diffuse intrinsic pontine gliomas (DIPGs) and thalamic gliomas, have very poor prognoses with a median survival of 8–11 months for DIPGs (1–3) and about 25 and 12 months for WHO grade 3 and 4 thalamic gliomas, respectively (3–5). This is attributed to difficulty of radical surgery (6) and resistance to temozolomide (TMZ) (3, 7).

Recent genetic studies have shown that up to 90% of DMGs have oncogenic histone gene mutations, resulting in a lysine -to -methionine substitution (H3K27M) in *H3F3A* gene encoding histone H3.3 protein or in *HIST1H3B* gene encoding histone H3.1 protein (3, 8–12). Epigenetic studies have shown that these histone gene mutations cause diffuse DNA hypomethylation (3, 13, 14).

The DNA-repair enzyme O6-methyl-guanine-DNA methyltransferase (MGMT) inhibits the killing of tumor cells by alkylating agents such as TMZ (15). MGMT transcription is epigenetically regulated. *MGMT* promoter methylation inhibits the transcription of MGMT, leading to the silencing of MGMT (3, 15, 16). Multiple studies have shown that *MGMT* promoter methylation is a predictive factor of response to TMZ (16, 17). New studies have shown that 97–100% of DMGs with H3K27M mutation lack *MGMT* promoter methylation (18, 19). Therefore, we can surmise that epigenetic changes driven by histone H3K27M mutations cause a frequent lack of *MGMT* promoter methylation, leading to increased expression of MGMT and resistance to TMZ therapy (3).

We set out to investigate this hypothesis in the preclinical setting using DMG cell lines. We established a cell line that has H3K27M mutation of *HIST1H3B*, the rarer form of histone H3K27M, from a diffuse intrinsic pontine glioma (DIPG) patient. Using this cell line and other DMG cell lines, we have demonstrated for the first time *in vitro* that MGMT expression contributes to resistance to TMZ in H3K27M mutant DMG cell lines.

## Materials and Methods

### Human Tissue Specimens

Human DIPG specimens were obtained during surgery in accordance with institutional review board approvals (Niigata University #2583) after obtaining written consent from the family.

### Immunohistochemistry and Pathological Diagnosis

The surgical specimens were fixed with 20% buffered formalin and embedded in paraffin. Histopathological examination was performed on 4-μm-thick sections stained with hematoxylin and eosin, and the paraffin-embedded sections were processed for immunohistochemistry using methods previously described (20, 21). The histological diagnosis was made in accordance with the World Health Organization (WHO) classification of tumors of the central nervous system (CNS) (22). Primary monoclonal antibodies against MGMT (MAB16200, Merck, Darmstadt, Germany; dilution 1:100) and histone H3K27M (ABE419, Merck; 1:500) were used.

### Establishment of a DMG Cell Line

**Figure 1:**
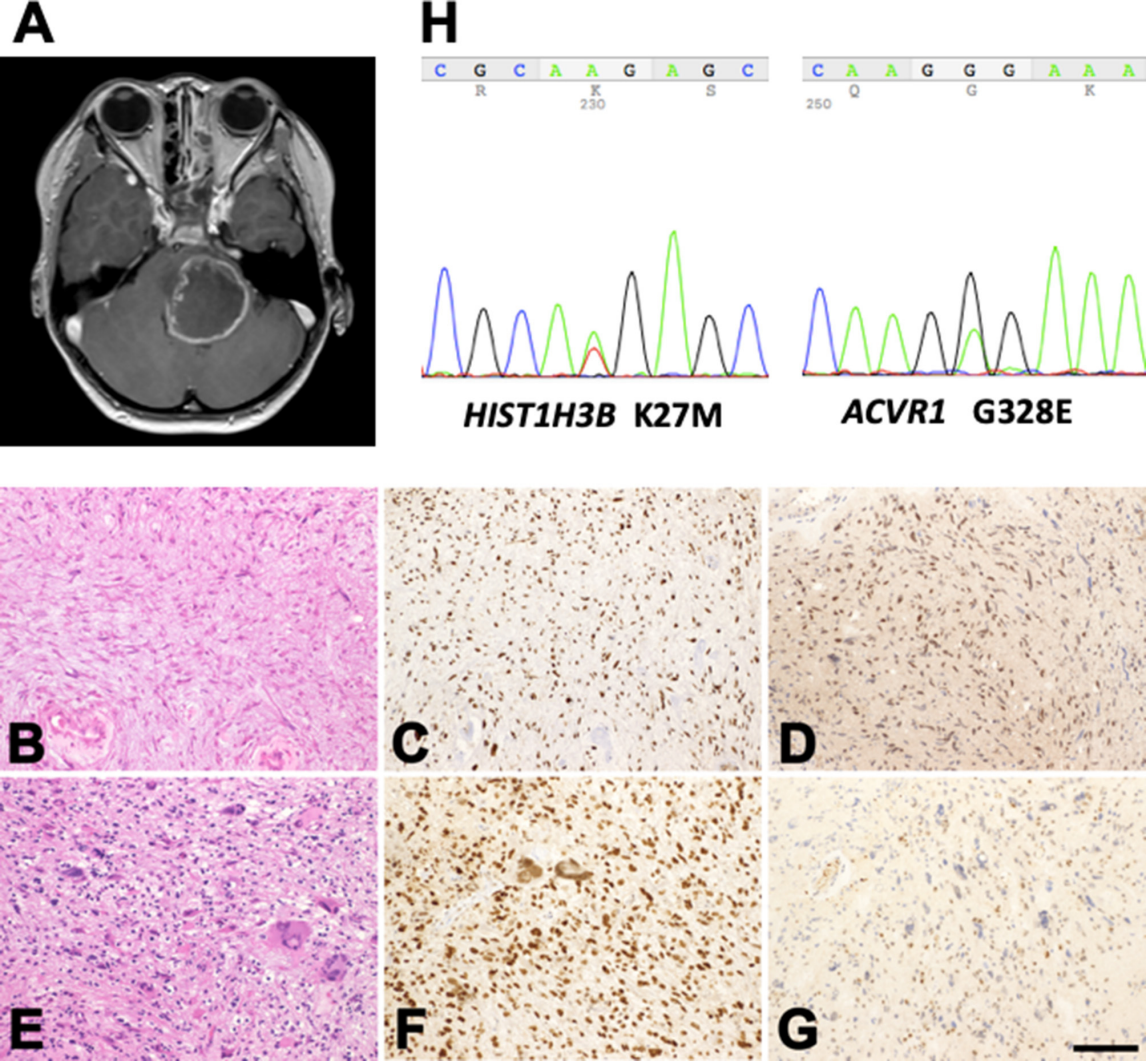
Profile of the patient with H3K27M-mutant diffuse midline glioma. **(A)** Post-contrast MR images disclose a large mass lesion involving the pons. **(B–G)** Histology and immunohistochemistry of the surgical specimens taken at the first **(B–D)** and second **(E–G)** operations. **(B)** Astrocytic tumor cells with fine processes. **(E)** Tumor cells with marked nuclear atypia. **(C,F)** Histone H3K27M-immunohistochemistry. A large proportion of the tumor cell nuclei in the specimens taken at both operations are positive. **(D,G)** MGMT-immunohistochemistry. The proportion of positive nuclei in the specimens at the first operation is large **(D)**, but that in the specimens at the second operation is small **(G)**. **(B,E)** Hematoxylin and eosin staining. Bar = 120 μm for **(B–G)**. **(H)** K27M mutation in *HIST1H3B* and G328E mutation in *ACVR1* in the tumor tissue removed during the second operation.

The NGT16 cell line was derived from surgical specimen taken from a DIPG patient (Figure 1A) during the second removal operation. The MR image has been used for the figure after obtaining consent from the parents. The specimen was minced with a scalpel and incubated in papain solution (Worthington Biochemical Corporation, Lakewood, NJ, USA) at 37°C for 30 min with shaking every few minutes to dissociate the tissue as previously described (23). The tissue was triturated using a sterile pipette until no clumps were visible. After centrifugation of the suspension, the cell pellets were washed with PBS and maintained in Dulbecco's modified Eagle medium (DMEM) (Thermo Fisher Scientific, Waltham, MA, USA) supplemented with 10% fetal bovine serum (FBS) (Sigma Aldrich, St. Louis, MO, USA) and 1% Antibiotic-Antimycotic (Thermo) (24). The cells were passaged before becoming confluent and by splitting 1:2 after detachment using trypsin (Thermo).

DIPG cell lines, SF7761 (25) and SF8628 (26), are a kind gift of Dr. Nalin Gupta (University of California, San Francisco, CA, USA), and JHH-DIPG1 is a kind gift of Drs. Eric H. Raabe and Charles G. Eberhart (Johns Hopkins University, Baltimore, MD, USA) (27). All three cell lines are known to have the H3.3 K27M mutation. Control glioblastoma (GBM) cell lines, U87MG and T98G, without and with MGMT protein expression, respectively (28, 29), were purchased from American Type Culture Collection (ATCC). All cell lines were grown in a humidified 37°C incubator at 5% CO_2_. NGT16, SF8628, U87MG, T98G cells were grown as adherent monolayer cultures in 10% FBS DMEM. SF7761, JHH-DIPG1 cells were grown as sphere cultures in EF20 medium composed with Neurobasal Medium (Thermo), 20 ng/ml EGF (Peprotech, Rocky Hill, NJ, USA), 20 ng/ml FGF (Peprotech), 2% B27 supplement (Thermo), 0.25% N2 supplement (Thermo), 3 mM L-Glutamine (Thermo), 2 μg/ml Heparin (Sigma), and 1% Antibiotic-Antimycotic (Thermo) (30, 31).

### DNA Extraction and Sequencing

We extracted DNA from cell lines by using Nucleospin tissue Kit (Takara Bio Inc., Shiga, Japan), and DNA from surgically obtained formalin-fixed paraffin embedded (FFPE) tissue by using QIAamp DNA FFPE Tissue Kit (Qiagen, Hilden, Germany) (23, 32). PCR was performed to amplify fragment of *H3F3A* gene or *HIST1H3B* gene including the mutational region of H3K27M. Primer sequences for the *H3F3A* were 5′- GAT TTT GGG TAG ACG TAA TCT TCA−3′ (forward) and 5′- TTT CCT GTT ATC CAT CTT TTT GTT−3′ (reverse) and for the *HIST1H3B* were 5′- GGG CAG GAG CCT CTC TTA AT−3′ (forward) and 5′- ACC AAG TAG GCC TCA CAA GC−3′ (reverse) as described previously (33). Polymerase chain reaction (PCR) products were electrophoresed on agarose gel and target DNA fragments were excised from the gel and purified by using Wizard SV Gel and PCR Clean-Up System (Promega Corporation, Madison, WI, USA) (23, 32). Purified DNA fragments were sent for Sanger sequencing at Eurofins Genomics (Tokyo, Japan).

### Methylation Specific PCR (MSP)

DNAs were subjected to bisulfite modification by using MethylEasy Xceed Rapid DNA Bisulphite Modification Kit (Takara Bio Inc.), and PCR was performed to amplify fragment of *MGMT* gene including CpG-rich promoter region. Primer sequences of *MGMT* were for the unmethylated reaction 5′- TTT GTG TTT TGA TGT TTG TAG GTT TTT GT−3′ (forward) and 5′- AAC TCC ACA CTC TTC CAA AAA CAA AAC A−3′ (reverse) and for the methylated reaction 5′- TTT CGA CGT TCG TAG GTT TTC GC−3′ (forward) and 5′- GCA CTC TTC CGA AAA CGA AAC G−3′ (reverse) as described previously (15).

### Western Blotting

Total cell lysate was collected from asynchronously proliferating cells in buffer (125 mM Tris-HCl (pH 6.8), 4% SDS, 20% Glycerol) containing protease inhibitors (1 mM EDTA, 20 μg/ml Leupeptin, 10 μg/ml Pepstatin, 1 mM PMSF) and phosphatase inhibitors (1 mM NaF, 1 mM Na_2_MoO_4_, 1 mM Sodium orthovanadate). Fifteen microgram of protein from each Lysate sample were separated by SDS-PAGE and transferred to polyvinylidene difluoride membranes (Merck). After probing with primary antibodies, the membranes were incubated with horseradish peroxide-conjugated secondary antibody [1:1,000; Cell Signaling Technology (CST), Danvers, MA, USA], and visualized by ECL prime Western Blotting Detection Reagent (GE Healthcare Life Sciences, Chicago, IL, USA) (23). Mouse monoclonal anti-MGMT antibody (1:500; MAB16200, Merck), rabbit monoclonal anti-β-actin antibody (1:2,000; #4970S, CST) were used.

### Cell Viability Assay

One thousand five hundred cells per 100 μl medium were incubated on a 96 plate for 1 day and applied 10 μl of TMZ (Sigma) dissolved in dimethyl sulfoxide (DMSO) at concentrations of 0, 62.5, 125, 250, and 500 μM and incubated for 72 h, and ALK2 inhibitor K02288 (Selleck, Houston, TX, USA) dissolved in DMSO at concentrations of 0, 12.5, 25, and 50 μM of. As a background control (0% control), TritonX-100 (final concentration 0.2%) was added to the control wells. Ten microliter of WST-8 (Nacalai Tesque, Kyoto, Japan) was added to each well, and the cells were incubated for 4 h. Absorbance at 450 nm was measured in each well using ELx808 (BioTek Instruments, Winooski, VT, USA) plate reader (23).

### 5-Methylcytosine (5-mC) Assay

MethylFlash global DNA methylation (5-mC) ELISA easy kit (EpiGentek, Farmingdale, NY, USA) was used to measure DNA methylation of cell lines. Briefly, 100 ng of DNA extracted from each cell line was placed in assay wells, washed then detection complex solution was added. After incubation, wells were washed and color developer solution was added to each well and absorbance was measured at 450 nm with ELx808 plate reader. Positive and negative controls were used to demonstrate a standard curve.

### Statistical Analysis

Differences between two or more groups were assessed using two-way ANOVA test with *post hoc* Tukey's multiple comparison test. Error bars represent standard error of means (SEM) (34). All statistical tests were performed using the GraphPad Prism 7 software (GraphPad Software, La Jolla, CA, USA). *P* < 0.05 was considered statistically significant.

## Results

### Clinical Features and Establishment of NGT16 Cell Line

Histologically, the tumor taken at the first operation showed astrocytic cells with fine processes and nuclear atypia (Figure 1B). Hyalinization of the vessel walls and tissue necrosis were evident. Immunohistochemically, the tumor cell nuclei were positive for histone H3K27M (Figure 1C) and MGMT (Figure 1D). The tumor taken at the second operation was consisted of high-cellular astrocytic cells with marked nuclear atypia and occasional multinucleated giant cells (Figure 1E). The cell nuclei were immunopositive for histone H3K27M (Figure 1F). However, only a small number of the cells exhibited MGMT-immunopositivity (Figure 1G), possibly indicating the effect of TMZ on the tumor cells. DNA sequencing of the tumor revealed the H3K27M mutation in *HIST1H3B* (Figure 1H, *left*), a rare mutation variant in histone, and G328E mutation in *ACVR1* (Figure 1H, *right*), a frequently encountered mutation in patients with DIPGs with *HIST1H3B* mutation (10, 33, 35). No H3K27M mutation in *H3F3A* was detected. The patient succumbed to death 18 months after initial presentation of her disease.

### *HIST1H3B* and *ACVR1* Mutations Are Present in the NGT16 Cell Line

**Figure 2:**
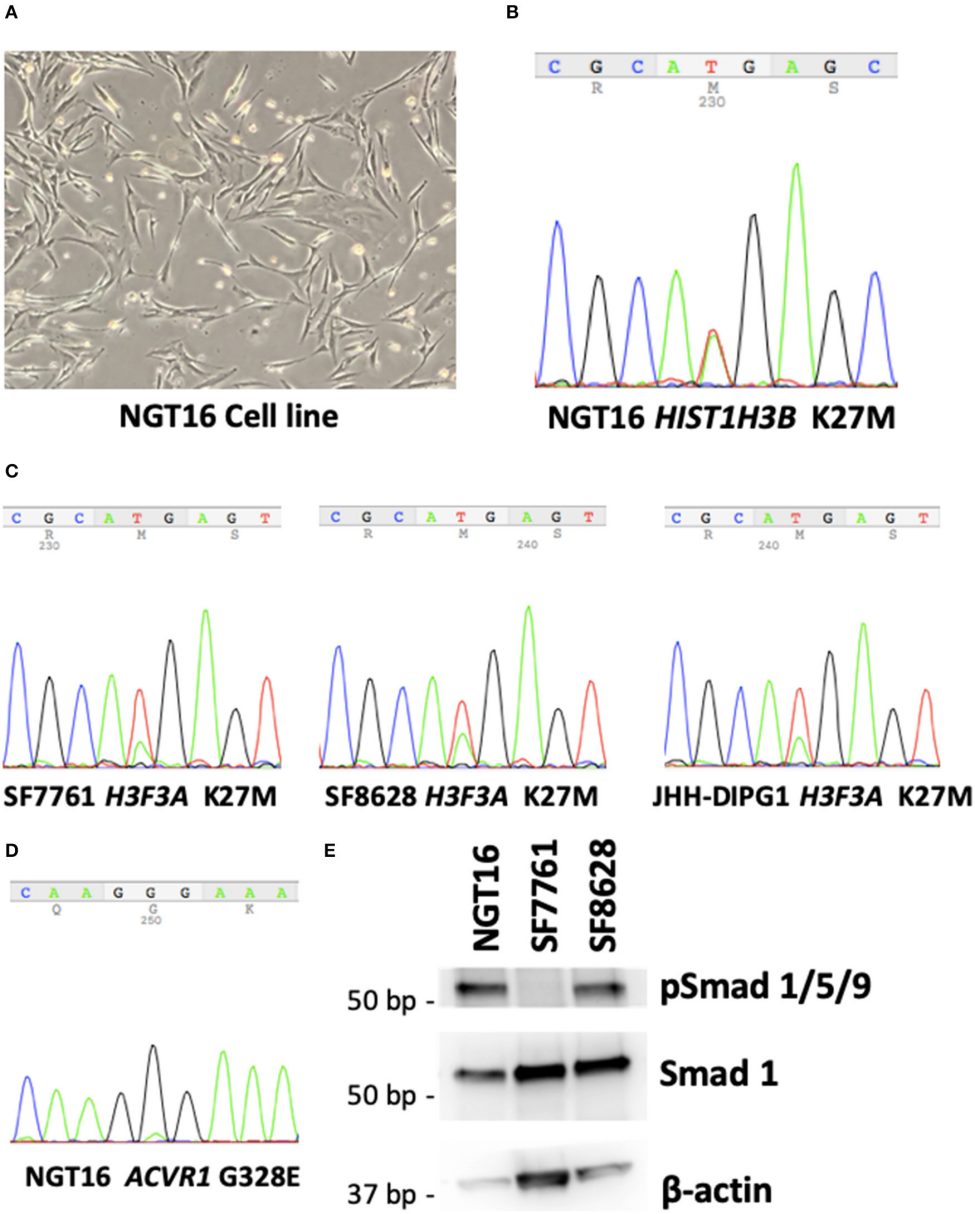
NGT16 harbors *HIST1H3B* K27M and *ACVR1* mutations. **(A)** NGT16 cell line grows as adherent monolayer in culture. **(B)** *HIST1H3B* K27M mutation was confirmed in NGT16. **(C)** *H3F3A* K27M mutations were verified in SF7761, SF8628, and JHH-DIPG1 cell lines. **(D)** *ACVR1* G328E mutation was also detected in NGT16, confirming that mutations found in the primary tumor were preserved in the cell line. **(E)** Downstream activation of the BMP pathway was observed in NGT16 and SF8628 cell lines, but not SF7761.

Having established a cell line (Figure 2A) from a DMG harboring mutations in *HIST1H3B* and *ACVR1* (Figure 1H), we set out to confirm whether the same mutations could be identified in the established cell line. Sanger sequencing of DNA taken from the NGT16 cell line showed H3K27M mutation of *HIST1H3B* (Figure 2B) and *ACVR1* (Figure 2D), confirming that mutations which were present in the primary tumor were also present in the cell line. The glutamic acid (E) peak for *ACVR1* was very small, suggesting that only a proportion of the cultured tumor cells harbored the *ACVR1* G328E mutation, consistent with data obtained from biopsy samples in a previously published study (35). The more common *H3F3A* mutation was confirmed in SF7761, SF8628, and JHH-DIPG1 cell lines (Figure 2C).

### BMP Pathway Is Activated in NGT16 Cell Line

Activin receptor type 1 (*ACVR1*) mutations are frequently found in HIST1H3B-mutant, but not H3.3 H3K27M-mutant DMGs (10, 36). *ACVR1* encodes for the type I bone morphogenic protein (BMP) receptor ALK2, and mutation of this receptor causes constitutive activation of BMP signaling pathway (36). Phosphorylation of downstream SMAD 1,5,9 was noted in NGT16 (Figure 2E), suggesting BMP pathway activation. An approximately 30% reduction in cell viability after 72-h treatment with 50 μM of ALK2 inhibitor K02288 was observed (Supplementary Figure 1), which is >100 μM level known to cause toxicity in normal cells (37). Interestingly, phospho-SMAD 1,5,9 was also expressed in SF8628 line lacking *ACVR1* mutation (Figure 2E), suggesting that the BMP pathway can be activated in DMGs in an *ACVR1* mutation independent manner, thus a potentially broader application of ALK2 inhibitors exists in DMGs.

### A Majority of DMG Cell Lines Lack Methylated MGMT Promoter

**Figure 3:**
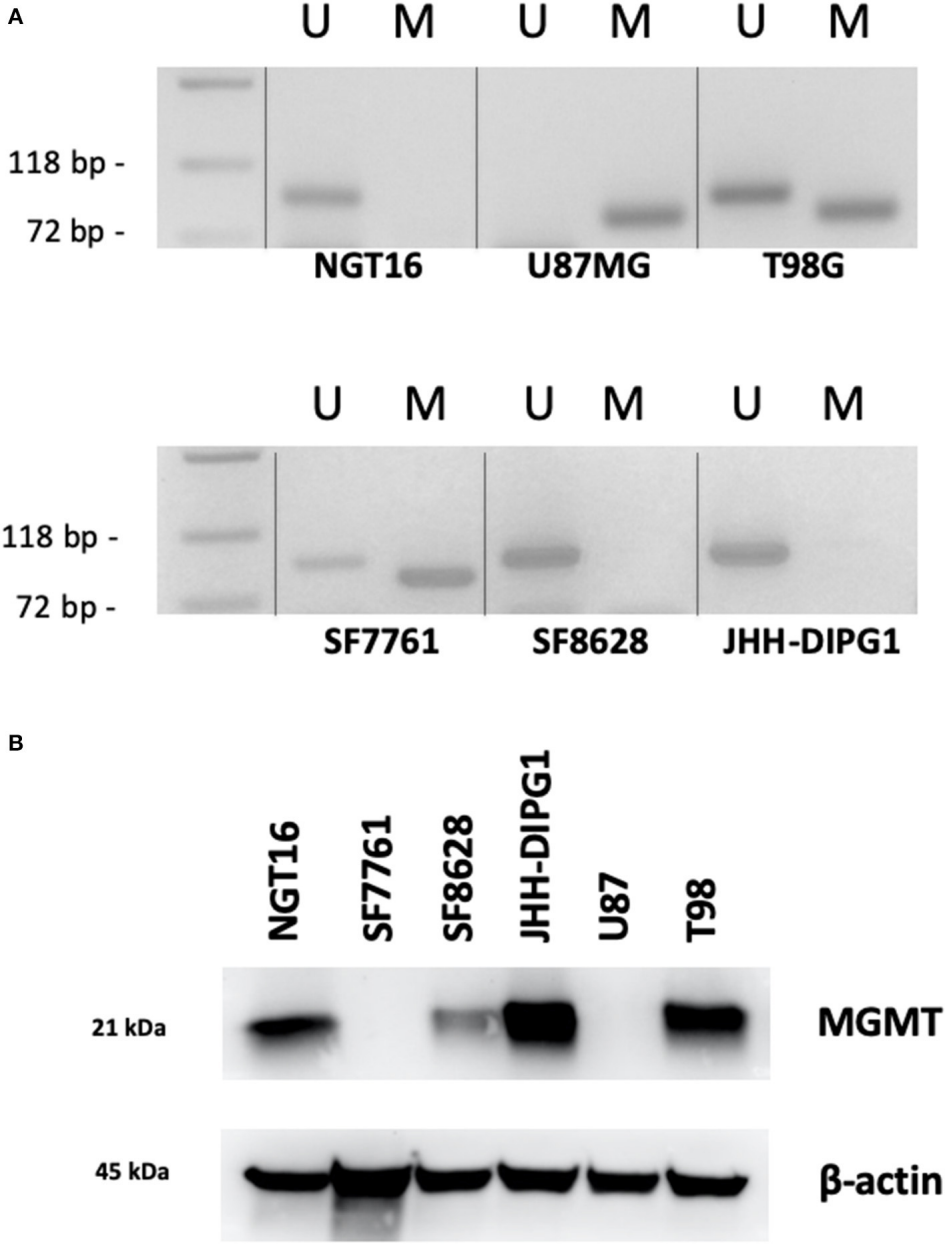
*MGMT* promoter methylation and MGMT expression in each cell line. **(A)** Methylation specific PCR (MSP) showed that of the H3K27M diffuse midline glioma (DMG) cell lines, NGT16, SF8628, and JHH-DIPG1 lines had unmethylated *MGMT* promoter, whereas in SF7761, both bands were seen, but the methylated (M) band was stronger. **(B)** Western blot shows MGMT expression in NGT16, SF8628, JHH-DIPG1 and T98G, but no expression in SF7761 and U87MG cell lines, consistent with results of MSP.

Next, we analyzed methylation level at the *MGMT* promoter of each cell line to determine whether *MGMT* promoters are unmethylated in DIPG cell lines harboring histone H3K27M as indicated in prior studies of *MGMT* promoter methylation in DMG tissues (18, 19). MSP for *MGMT* promoter of each cell line was conducted to obtain methylated (M) and unmethylated (U) bands. The GBM control cell line U87MG showed only the M band whereas T98G cells showed both M and U bands (Figure 3A). This data was consistent with a previously published report (38). H3K27M mutant DMG cell lines NGT16, SF8628, JHH-DIPG1 showed only the U band, whereas SF7761 showed both U and M bands, the M band being stronger. Collectively, in 3 out of 4 (75%) DMG cell lines, including NGT16, the *MGMT* promoter was unmethylated.

### MGMT Protein Expression Is Elevated in DIPG Cell Lines With Unmethylated *MGMT* Promoter

Having confirmed the *MGMT* promoter status in GBM and DMG cell lines, we next sought to analyze MGMT protein expression levels in each cell line by Western blotting. MGMT expression was negative for U87MG and positive for T98G as known previously (28, 29). MGMT expression was positive for H3K27M mutant DMG cell lines NGT16, SF8628, JHH-DIPG1 but negative for SF7761, in exact agreement with the results of MSP (Figure 3B).

### DMG Cell Lines With MGMT Expression Are Resistant to TMZ *in vitro*

**Figure 4:**
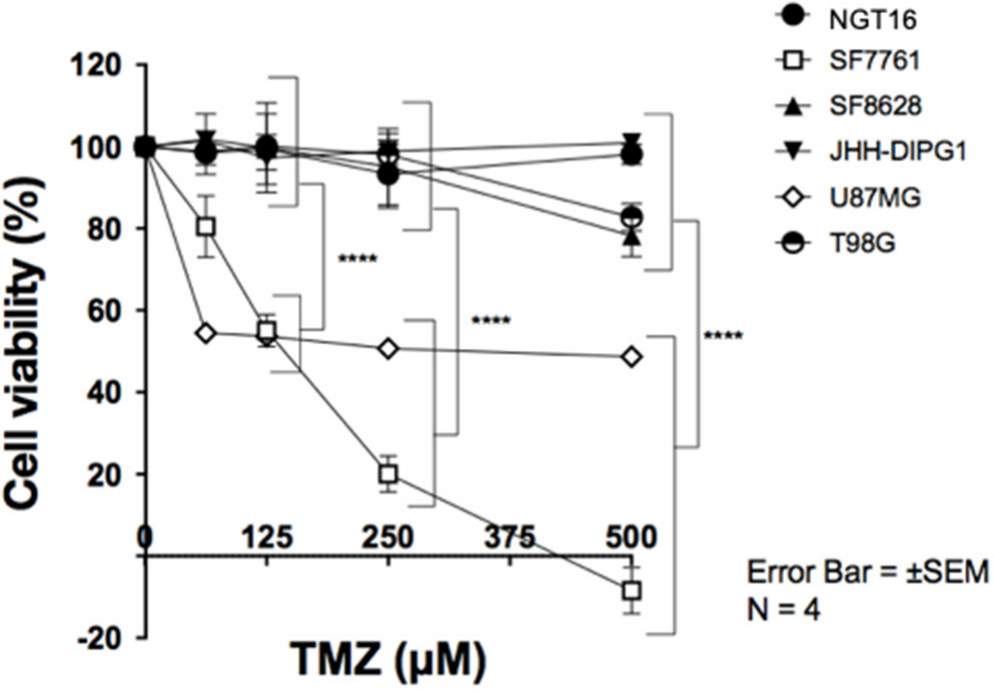
SF7761 and U87MG are sensitive to temozolomide (TMZ). Cell viability assays showed that cell lines with reduced MGMT expression, SF7761 and U87MG, are sensitive to TMZ, whereas NGT16, SF8628, JHH-DIPG1, and T98G lines, which retain MGMT expression, were resistant to TMZ (^⋆⋆⋆⋆^*p* < 0.0001; Two-way ANOVA).

Finally, we evaluated the sensitivity of DMG cell lines to TMZ in association with *MGMT* promoter methylation and MGMT expression. DMG cell lines were treated with TMZ at concentrations of 0, 62.5, 125, 250, and 500 μM for 72 h and cell viability was determined using WST-8 assay. MGMT expressing cell lines, NGT16, SF8628, JHH-DIPG1, and T98G, demonstrated resistance to TMZ uniformly. In contrast, SF7761 and U87MG, which did not express MGMT protein, showed marked sensitivity to TMZ (Figure 4, *p* < 0.0001 at 125–500 μM TMZ compared to cell viability of NGT16, SF8628, JHH-DIPG1, and T98G). IC50 was 403.5 μM and 206.6 μM for U87MG and SF7761, respectively. Presence of histone mutations, *MGMT* promoter methylation, MGMT protein expression and sensitivity to TMZ for the 6 cell lines are shown in Table 1.

**Table 1:** Characteristics of each cell line are summarized.

**Cell line**	**Histology**	**Histone H3 mutation**	**MGMT promoter**	**MGMT** **expression**	**TMZ resistance**
NGT16	DIPG	H3.1 K27M	Unmethylated	+	+
SF7761	DIPG	H3.3 K27M	Partially methylated	–	–
SF8628	DIPG	H3.3 K27M	Unmethylated	+	+
JHH-DIPG1	DIPG	H3.3 K27M	Unmethylated	+	+
U87MG	GBM	-	Methylated	–	–
T98G	GBM	-	Unmethylated	+	+

*M, methylated MGMT promoter; MSP, methylation specific PCR; U, unmethylated MGMT promoter*.

### DNA Hypomethylation Is Observed in NGT16 Cell Line

To determine the global DNA methylation changes of each cell line, levels of 5-methylcytosine (5-mC) were assessed in NGT16, SF7761, SF8628, JHH-DIPG1, T98G, and U87MG cell lines. DNA methylation occurs by the covalent addition of a methyl group at the 5-carbon of the cytosine ring by DNA methyltransferases, resulting in 5-mC. Thus, global DNA methylation can be analyzed by determining 5-mC levels. Although not all H3K27M mutant cell lines showed decreased 5-mC expression compared to wildtype cell lines (Table 2), NGT16 cell line expressed the least 5-mC (5-mC/(5-mC+C) = 0.44%). On the other hand, SF7761, whose *MGMT* promoter was methylated and was sensitive to TMZ, showed highest percentage of 5-mC of all cell lines (5-mC/(5-mC+C) = 2.63%).

**Table 2:** 5-methylcytosine (5-mC) levels of each cell line is listed.

**Cell line**	**NGT16**	**SF7761**	**SF8628**	**JHH-DIPG1**	**T98G**	**U87MG**
5-mC/(5-mC+C) (%)	0.44	2.63	1.82	2.08	2.31	0.77

*DNA of SF7761 cell line was hypermethylated. 5-mC, 5-methylcytosine; C, cytosine*.

## Discussion

**Figure 5:**
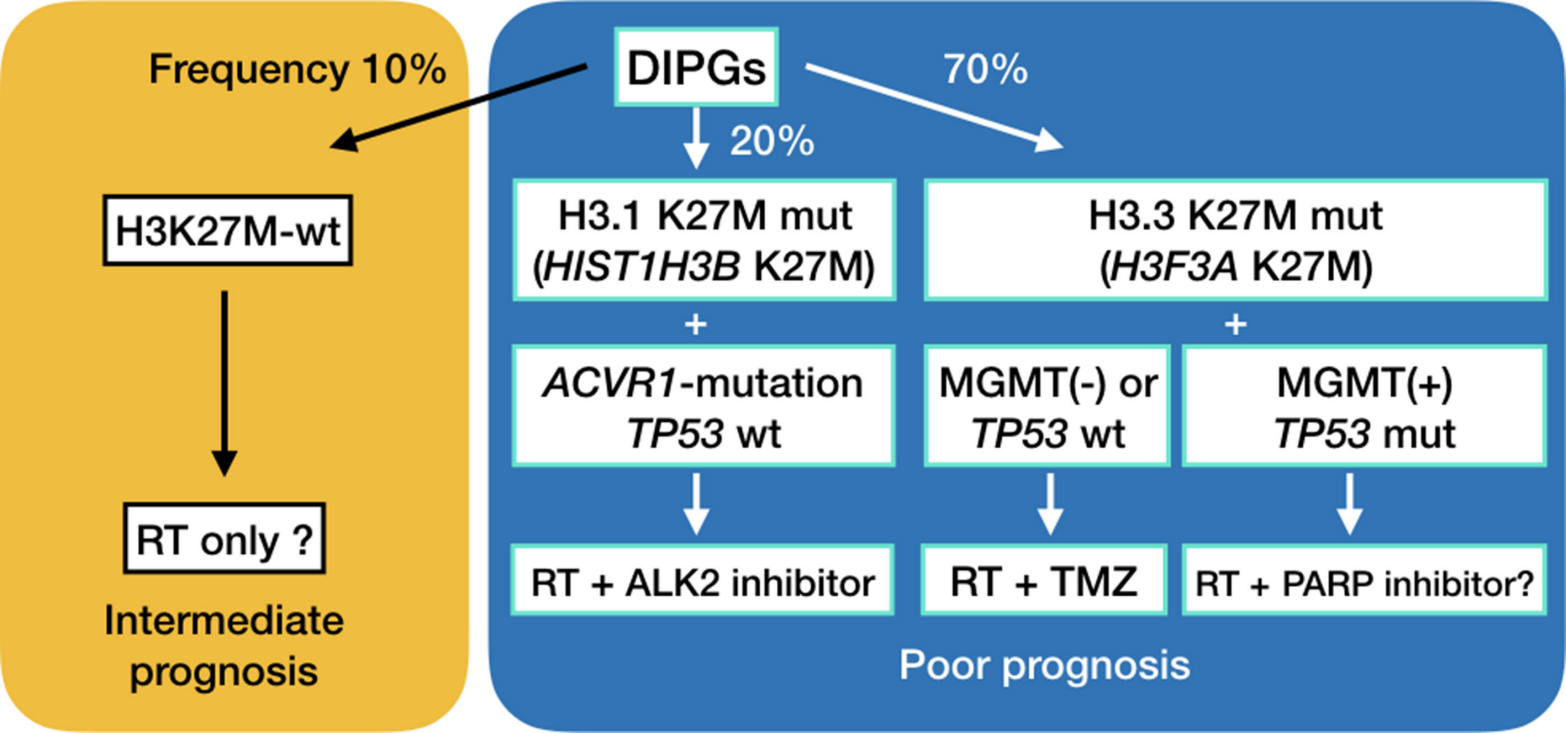
Suggested treatment for diffuse midline gliomas.

Recent genetic studies have shown that up to 90% of DMGs have H3K27M mutations in *H3F3A* encoding histone H3.3 or in *HIST1H3B* encoding histone H3.1 (8–12). H3.3 H3K27M mutations are about 2.5-fold more frequent, present at an older age, occur more frequently in boys, are less sensitive to radiation treatment and carry a worse prognosis compared to DIPGs harboring H3.1 H3K27M mutations (3, 10). Interestingly, *ACVR1* mutations frequently are found as a secondary hit in DIPG with H3.1 H3K27M mutations, as opposed to frequent chromosomal abnormalities and *TP53* mutations in DIPGs with H3.3 H3K27M mutations (Figure 5) (10, 36, 39). We established a DIPG cell line harboring the H3.1 H3K27M and *ACVR1* G328E mutations. BMP pathway activation was observed in the cell line.

Epigenetic studies have shown that histone mutations cause DNA hypomethylation (3, 13, 14). Recent studies have shown that DMGs with histone mutations frequently lack MGMT promoter methylation. In a study of 46 DMGs with confirmed *H3F3A* mutation, none had *MGMT* promoter methylation (19). Similarly, a different study showed *MGMT* promoter was methylated in only 3% of DIPGs with H3K27M mutations (18). Also, an immunohistochemical analysis of MGMT revealed positive staining in 9 out of 11 (82%) brainstem gliomas (40). Indeed, it can be hypothesized that epigenetic changes triggered by histone mutation cause MGMT to be expressed in most DMGs, and thus render them resistant to TMZ. In the present study, 3 out of 4 DMG cell lines (NGT16, SF8628, and JHH-DIPG1) having unmethylated *MGMT* promoter and MGMT expression were resistant to TMZ. One cell line, SF7761, had a methylated *MGMT* promoter, lacked MGMT protein expression, and was sensitive to TMZ treatment. Epigenetic regulation of MGMT in H3K27M-mutant DMGs have been outlined in a recent review we published (3).

Previous reports looking at TMZ sensitivity in GBM cell lines have suggested that TMZ sensitivity correlated with not only MGMT, but also p53 status (29). Wildtype p53 enhances the apoptotic effect of temozolomide (29) via Fas/CD95/Apo-1 receptor activation, whereas in mutant p53 the mitochondrial apoptotic pathway via Bcl-2 degradation is triggered, which is significantly less effective (41). A recent review article elegantly details the differences in DNA damage and subsequent DNA repair triggered by temozolomide and nitrosorueas (42). In one study, only 2 out of 12 GBM cell lines (T98G and LN-18) had elevated MGMT protein expression, indicating MGMT expression may be lost during culturing of GBM cell lines (29). However, in the present study, MGMT expression was maintained in all 3 DMG cell lines with unmethylated *MGMT* promoter. We did not look at p53 status in the cell lines of the present study, as TMZ sensitivity correlated well with MGMT status. Previous studies have shown that T98G and SF8628 are p53-mutant, whereas U87MG and SF7761 are p53-wildtype (26). It is important to note that TP53 accumulation was found in 16 out of 26 (61.5%) H3.3 H3K27M-mutant DMGs, but 0 out of 12 (0%) H3.1 H3K27M-mutant DMGs (10).

## Future Directions

We have previously outlined existing preclinical and clinical trials in DMGs (3, 43), including epigenetic modification, mTOR inhibition, immunotherapy and convection-enhanced delivery. DMGs are generally not amenable to radical surgery, and are not sensitive to TMZ, the first line chemotherapy treatment in adult glioblastomas, as shown in the present study. TMZ is known to methylate adenine at N3-position and guanine at N7-position in addition to O6-position of guanine. The first two usually do not induce cytotoxicity, because poly (ADP-ribose) polymerase (PARP) activation allows for base excision repair of damaged DNA. Inhibition of PARP or depletion of NAD^+^, which is a co-enzyme of PARP, leads to cell death (3, 44). A previous study has shown PARP1 expression in DIPG cell lines (45). PARP inhibition in conjunction with a DNA damaging treatment such as radiation is a potential treatment combination for MGMT-expressing, *TP53*-mutant diffuse midline gliomas (Figure 5). We have looked at the sensitivity of DMG cell lines to PARP inhibitors, and have found that sensitivity is very cell line specific (unpublished data). Furthermore, *in vivo* studies are needed to be done to test the efficacy of temozolomide and PARP inhibitors in these cell lines. Finding the determining factor of PARP sensitivity may be a key to finding a cure for this deadly disease.

Furthermore, there is excitement in the research community that ALK2 inhibition may be effective in *ACVR1*-mutant DMGs. Data from the present research suggests that BMP pathway can be activated in *ACVR1*-wildtype DMGs as well. Development of multiple DMG cell lines which harbor *ACVR1* mutation is necessary for further research (39).

The suggested treatments for diffuse midline gliomas are outlined in Figure 5. Initial biopsy of diffuse midline gliomas, including diffuse intrinsic pontine gliomas, will be needed to assess the optimal method of treatment. Convection enhanced delivery has also been explored in these deadly tumors (46–49), and surgical access of these lesions is expected to become more and more important (50).

## Conclusion

Efforts to improve the prognosis of DMGs have been largely unsuccessful. One reason is that DMGs are not sensitive to TMZ, which is a key drug in the treatment of GBM. We confirmed through *in vitro* studies that epigenetically driven high expression of MGMT is the main reason for TMZ resistance.

## Data Availability Statement

The datasets analyzed during the current study are available from the corresponding author upon request.

## Ethics Statement

The studies involving human participants were reviewed and approved by Niigata University Institutional Review Board. Written informed consent to participate in this study was provided by the participants' legal guardian/next of kin. Written informed consent was obtained from the minor(s)' legal guardian/next of kin for the publication of any potentially identifiable images or data included in this article.

## Author Contributions

HA, MOk, RH, and MN designed the study. HA, MOk, JW, and YK performed experiments. YK and AK performed pathological evaluation. YT, JW, MN, JY, and MOi provided patient care and obtained written consent. YF approved the study design. All authors read and approved the final manuscript.

### Conflict of Interest

The authors declare that the research was conducted in the absence of any commercial or financial relationships that could be construed as a potential conflict of interest.

## Supplementary Material

Supplementary Figure 1 A modest 30% reduction in cell viability after 72-h treatment with 50 μM of ALK2 inhibitor K02288 was observed.
