# Supplementary figures and images for: MGMT Expression Contributes to Temozolomide Resistance in H3K27M-Mutant Diffuse Midline Gliomas
Source: Front Oncol. 2020 Jan 21;9:1568. doi: 10.3389/fonc.2019.01568 (PMC6985080; doi:10.3389/fonc.2019.01568)

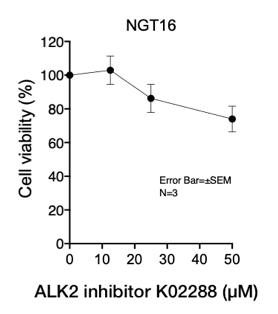

Supplement: Supplementary Figure 1 — A modest 30% reduction in cell viability after 72-h treatment with 50 μM of ALK2 inhibitor K02288 was observed. [file Image_1.TIF]
